# Chloridotris[μ_2_-2-(dimethyl­amino)­ethano­lato]-μ_3_-hydroxido-tri-μ_2_-trifluoro­acetato-tetra­copper(II) tetra­hydro­furan solvate

**DOI:** 10.1107/S1600536810022877

**Published:** 2010-06-18

**Authors:** S. Tajammul Hussain, Shahzad Abu Bakar, Mohammad Mazhar, Matthias Zeller

**Affiliations:** a National Center for Physics, Quaid-i-Azam University, Islamabad 45320, Pakistan; b Department of Chemistry, Faculty of Science, University of Malaya, Lembah Pantai, 50603-Kuala Lumpur, Malaysia; c STaRBURSTT Cyber Instrumentation Consortium, Youngstown State Unversity, Department of Chemistry, One University Plaza, Youngstown, Ohio 44555-3663, USA

## Abstract

The title compound, [Cu_4_(C_2_F_3_O_2_)_3_(C_4_H_10_NO)_3_Cl(OH)]·C_4_H_8_O or [Cu_4_(TFA)_3_(dmae)_3_Cl(OH)]·THF (dmae is dimeth­yl­amino­ethano­late, TFA is trifluoro­acetate and THF is tetra­hydro­furan), has an approximate mol­ecular threefold symmetry with three equivalent {Cu(dmae)(TFA)} units bridging between a Cu—Cl and a hydroxide unit, with the latter two lying on the mol­ecular threefold axis. However, in the solid state, the tetranuclear complex has *C_i_* symmetry. The Cu atom bonded to the Cl atom has a distorted tetra­hedral geometry. The other three Cu atoms have distorted square-pyramidal geometries with an NO_4_ coordination environment. The bonds within the CuNO_3_ base of the pyramid range from 1.953 (2) to 2.033 (3) Å, while the apical Cu—O bonds are significantly longer, ranging from 2.286 (2) to 2.377 (2) Å. The square-pyramidal geometries are augmented by weak inter­actions towards a sixth O atom, forming a highly distorted octa­hedral coordination environment [long Cu—O distances = 2.712 (2)–2.824 (2) Å]. The hydroxide group is hydrogen bonded to the tetra­hydro­furan solvent mol­ecule. One of the –CF_3_ groups shows minor disorder over two positions, with a refined occupancy ratio of 0.894 (4):0.106 (5).

## Related literature

For the synthesis of [Cu(dmae)Cl]_4_, used as starting material for title compound, see: Anwander *et al.* (1997 ▶). For general background to copper(II) complexes, see: Coastamagna *et al.* (1992 ▶). For related structures, see: Tahir *et al.* (2008 ▶); Shahid *et al.* (2009 ▶).
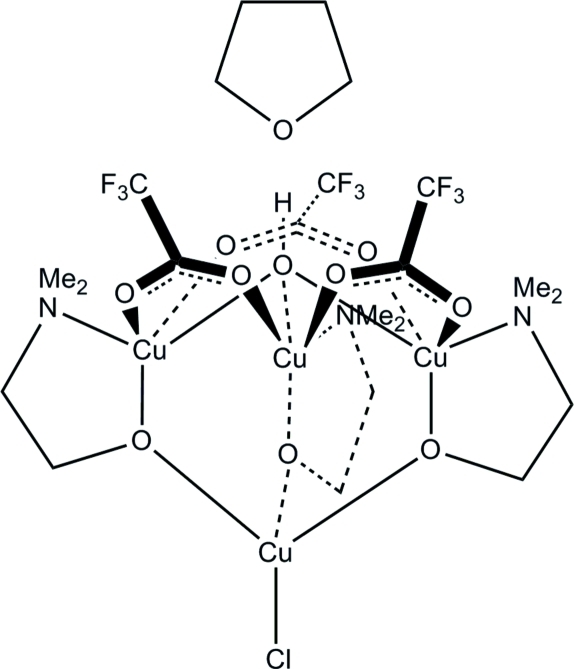


## Experimental

### 

#### Crystal data

[Cu_4_(C_2_F_3_O_2_)_3_(C_4_H_10_NO)_3_Cl(OH)]·C_4_H_8_O
*M* *_r_* = 982.21
Monoclinic, 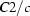

*a* = 16.4353 (14) Å
*b* = 12.1893 (12) Å
*c* = 35.547 (3) Å
β = 94.678 (2)°
*V* = 7097.7 (11) Å^3^
*Z* = 8
Mo *K*α radiation
μ = 2.54 mm^−1^
*T* = 100 K
0.41 × 0.38 × 0.28 mm

#### Data collection

Bruker SMART APEX CCD diffractometer
Absorption correction: multi-scan (*SADABS*; Bruker, 2008 ▶) *T*_min_ = 0.673, *T*_max_ = 0.746
20465 measured reflections
10267 independent reflections
7515 reflections with *I* > 2σ(*I*)
*R*_int_ = 0.035

#### Refinement

*R*[*F* ^2^ > 2σ(*F* ^2^)] = 0.043
*wR*(*F* ^2^) = 0.103
*S* = 1.01
10267 reflections
467 parameters
15 restraints
H-atom parameters constrained
Δρ_max_ = 0.92 e Å^−3^
Δρ_min_ = −0.69 e Å^−3^

### 

Data collection: *APEX2* (Bruker, 2008 ▶); cell refinement: *SAINT* (Bruker, 2008 ▶); data reduction: *SAINT*; program(s) used to solve structure: *SHELXTL* (Sheldrick, 2008 ▶); program(s) used to refine structure: *SHELXTL*; molecular graphics: *Mercury* (Macrae *et al.*, 2008 ▶); software used to prepare material for publication: *SHELXTL* and *publCIF* (Westrip, 2010 ▶).

## Supplementary Material

Crystal structure: contains datablocks I, global. DOI: 10.1107/S1600536810022877/fj2302sup1.cif

Structure factors: contains datablocks I. DOI: 10.1107/S1600536810022877/fj2302Isup2.hkl

Additional supplementary materials:  crystallographic information; 3D view; checkCIF report

## Figures and Tables

**Table 1:** Hydrogen-bond geometry (Å, °)

*D*—H⋯*A*	*D*—H	H⋯*A*	*D*⋯*A*	*D*—H⋯*A*
O10—H10⋯O11	1.00	1.73	2.723 (3)	174

**Fig. 1.**
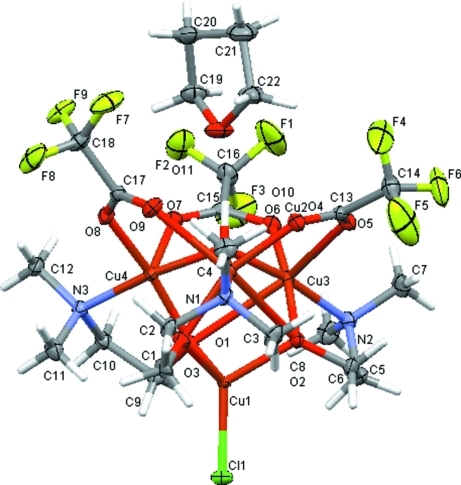
Perspective view of the title compound with the atom numbering scheme. The displacement ellipsoids are at the 50% probability level and H atoms are drawn as small spheres of arbitrary radii.

**Fig. 2.**
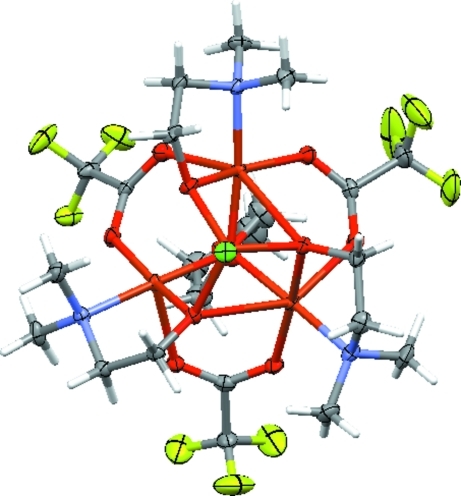
Perspective view of the title compound, view down the pseudo three fold axis. The displacement ellipsoids are at the 50% probability level and H atoms are drawn as small spheres of arbitrary radii.

## supplementary crystallographic information

### Comment

In recent years, there has been a considerable interest towards the synthesis of copper complexes; these complexes are extensively used in catalysis, enzymatic reactions, magnetism and molecular architecture (Coastamagna, Vargas *et al.*, 1992). The present work is a continuation of earlier studies for the preparation and structure eludication of copper (II) complexes (Shahid *et al.*, 2009). The motivation behind the synthesis of the title compound was to use it as a starting material for the synthesis of single source precursors for the deposition of thin films of copper oxides using aerosol assisted chemical vapor deposition (AACVD). We present here the synthesis and crystal structure of the title compound, [Cu_4_((CH_3_)_2_NCH_2_CH_2_O)_3_(F_3_CCOO)_3_(OH)Cl] or [Cu_4_(dmae)_3_(TFA)_3_(OH)Cl] (dmae = dimethylaminoethanolate, TFA = trifluoroacetate), which crystallized from THF as the mono solvate with the THF molecule tightly hydrogen bonded to the hydroxyl group.

The title compound has a slightly distorted molecular three fold symmetry with three equivalent Cu(dmae)(TFA) units bridging *via* their alcoholate oxygen atoms between a Cu—Cl and an hydroxyl unit with the latter two lying on a molecular pseudo threefold axis. The Cu atom bonded to the chlorine has a distorted tetrahedral geometry. The other three copper atoms have distorted square pyramidal geometries with a CNO_4_ coordination environment from the dmae O and N atoms, the hydroxyl O atom and two TFA anions. The TFA anions are bridging between two neighboring copper ions with one of the oxygen atoms being part of the base of the pyramid of one copper ion, and the other being in the apical position of the neighboring copper ion. The bonds within the CuNO_3_ bases of the pyramids are strong and quite similar in length with distances between 1.953 (2) and 2.033 (3) Å. The apical Cu—O bonds are significantly longer and between 2.286 (2) and 2.377 (2) Å, thus rendering the µ_2_-bridge of the TFA ions asymmetric. The square pyramidal geometries are augmented by weak interactions towards a fifth oxygen atom to form a highly distorted octahedral coordination environment (Cu—O distances: O3—Cu3 = 2.712 (2), O2—Cu2 = 2.780 (2), O1—Cu4 = 2.8240 (2) Å).

A similar motif as in the title compound was previously observed for two mixed metal copper-titanium complexes (Tahir *et al.*, 2008). In these complexes the TFA anions were replaced by benzoate or 2-methyl-benzoate ligands, and the Cu—Cl unit was replaced by a titanium atom, which in turn was bonded to another larger Cu—Ti cluster. The [Cu_3_(dmae)_3_(TFA)_3_(OH)] unit in the title compound and the [Cu_3_((CH_3_)_2_NCH_2_CH_2_O)_3_(O_2_C—C_6_H_5_R)_3_(OH)] units in the Cu—Ti complexes (*R* = H, Me) are quite similar. In the 2-methyl-benzoate complexes the [Cu_3_((CH_3_)_2_NCH_2_CH_2_O)_3_(O_2_C—C_6_H_5_Me)_3_(OH)] unit is located on an actual crystallographic three fold axis. The carboxylate anions show coordination modes differing slightly from those observed in the title compound with some of the oxygen atoms being detached from the copper ions and interactions to the fifth oxygen atom, which are very weak in the title compound, being strengthened instead. The overall coordination environment - distorted square pyramidal CNO_4 _geometries with an additional weak interaction towards a fifth oxygen atom - is however the same in all three compounds, which shows the idiosyncracy commonly observed for copper(II) to form strongly distorted and highly flexible octahedral geometries with a set of four strong bonds in a square planar arrangement and two apical ligands at variable distances. Individual ligand atoms in these kinds of complexes can easily switch from tightly bound to only weakly coordinated as long as the overall coordination environment of the metal center is retained, and energy differences and activation barriers between the different arrangements that can be achieved that way are quite small. The difference in bonding arrangement in the three complexes in the solid state does thus probably not translate into a different chemical nature for the three complexes as the bonding environment around Cu(II) is very flexible and it can be assumed that in solution (*i.e.* upon release of packing effects) all complexes will attain the same connectivity pattern.

In the title compound the hydroxyl group is O—H···O hydrogen bonded to a tetrahydrofuran molecule (Table 1), which is embedded in a bowl shaped cleft of the complex formed by the three TFA ligands. No such host–guest behavior was observed for the other two related compounds (Tahir *et al.*, 2008).

### Experimental

Tetrameric *N*,*N*-dimethylaminoethanolato copper(II) chloride, [Cu(dmae)Cl]_4_ was prepared according to a literature method (Anwander *et al.*, 1997). The title compound was prepared as follows: 1.25 g (1.67 mmole) of [Cu(dmae)Cl]_4_ in 20 ml THF were combined with 1.77 g (6.66 mmole) of Cu(F_3_CCOO)_2_ in 10 ml THF followed by the addition of 0.297 g (3.33 mmole) *N*,*N*-dimethylaminoethanol. The reaction mixture was stirred for 3 h and filtered through a cannula to remove any undissolved species. The filtrate was evaporated to dryness under vacuum, the solid was re-dissolved in 5 ml THF and placed in a vial with rubber seal at room temperature for one week to give blue crystals suitable for single-crystal X-ray diffraction analysis. Yield: 86% m.p. 393–394 K. Elemental Analysis for Cu_4_((CH_3_)_2_NCH_2_CH_2_O)_3_(F_3_CCOO)_3_(OH)Cl % calc: C, 21.99 H, 3.97 N, 4.27, % found: C, 22.10 H, 3.90 N, 4.53.

### Refinement

The fluorine atoms bonded to C14 were refined as disordered over two mutually exclusive positions with a refined occupancy ratio of 0.894 (4) to 0.106 (5). C—F bond distances within this CF_3_ group were restrained to be the same within a standard uncertaincy of 0.02 Å and ADPs of the minor F atoms were constrained to be identical to those of the major moiety F atom opposite their position.

All hydrogen atoms were added in calculated positions with a C—H bond distances of 0.97 (methylene), 0.96 (methyl) and 1.00 Å (OH). They were refined with isotropic displacement parameteres *U*_iso_ of 1.5 (methyl, OH) or 1.2 times *U*_eq_ (methylene) of the adjacent carbon or oxygen atom.

### Crystal data

**Table:**

[Cu_4_(C_2_F_3_O_2_)_3_(C_4_H_10_NO)_3_Cl(OH)]·C_4_H_8_O	*F*(000) = 3952
*M**_r_* = 982.21	*D*_x_ = 1.838 Mg m^−^^3^
Monoclinic, *C*2/*c*	Mo *K*α radiation, λ = 0.71073 Å
Hall symbol: -C 2yc	Cell parameters from 1722 reflections
*a* = 16.4353 (14) Å	θ = 2.4–30.1°
*b* = 12.1893 (12) Å	µ = 2.54 mm^−^^1^
*c* = 35.547 (3) Å	*T* = 100 K
β = 94.678 (2)°	Block, blue
*V* = 7097.7 (11) Å^3^	0.41 × 0.38 × 0.28 mm
*Z* = 8	

### Data collection

**Table:**

Bruker SMART APEX CCD diffractometer	10267 independent reflections
Radiation source: fine-focus sealed tube	7515 reflections with *I* > 2σ(*I*)
graphite	*R*_int_ = 0.035
ω scans	θ_max_ = 31.6°, θ_min_ = 1.2°
Absorption correction: multi-scan (*SADABS*; Bruker, 2008)	*h* = −12→22
*T*_min_ = 0.673, *T*_max_ = 0.746	*k* = −15→17
20465 measured reflections	*l* = −37→50

### Refinement

**Table:**

Refinement on *F*^2^	Primary atom site location: structure-invariant direct methods
Least-squares matrix: full	Secondary atom site location: difference Fourier map
*R*[*F*^2^ > 2σ(*F*^2^)] = 0.043	Hydrogen site location: inferred from neighbouring sites
*wR*(*F*^2^) = 0.103	H-atom parameters constrained
*S* = 1.01	*w* = 1/[σ^2^(*F*_o_^2^) + (0.0396*P*)^2^ + 12.2423*P*] where *P* = (*F*_o_^2^ + 2*F*_c_^2^)/3
10267 reflections	(Δ/σ)_max_ = 0.001
467 parameters	Δρ_max_ = 0.92 e Å^−^^3^
15 restraints	Δρ_min_ = −0.69 e Å^−^^3^

### Special details

**Table:**

Experimental. The fluorine atoms bonded to C14 were refined as disordered over two mutually exclusive positions with a refined occupancy ratio of 0.894 (4) to 0.106 (5). C-f bond distances within this CF3 group were restrained to be the same within a standard uncertaincy of 0.02 Angstrom and ADPs of the minor F atoms were constrained to be identical to those of the major moiety F atom opposite their position.
Geometry. All e.s.d.'s (except the e.s.d. in the dihedral angle between two l.s. planes) are estimated using the full covariance matrix. The cell e.s.d.'s are taken into account individually in the estimation of e.s.d.'s in distances, angles and torsion angles; correlations between e.s.d.'s in cell parameters are only used when they are defined by crystal symmetry. An approximate (isotropic) treatment of cell e.s.d.'s is used for estimating e.s.d.'s involving l.s. planes.
Refinement. Refinement of *F*^2^ against ALL reflections. The weighted *R*-factor *wR* and goodness of fit *S* are based on *F*^2^, conventional *R*-factors *R* are based on *F*, with *F* set to zero for negative *F*^2^. The threshold expression of *F*^2^ > σ(*F*^2^) is used only for calculating *R*-factors(gt) *etc*. and is not relevant to the choice of reflections for refinement. *R*-factors based on *F*^2^ are statistically about twice as large as those based on *F*, and *R*- factors based on ALL data will be even larger.

### Fractional atomic coordinates and isotropic or equivalent isotropic displacement parameters (Å^2^)

**Table:**

	*x*	*y*	*z*	*U*_iso_*/*U*_eq_	Occ. (<1)
C1	0.3487 (2)	0.6333 (3)	0.27202 (8)	0.0213 (7)	
H1A	0.2955	0.6699	0.2659	0.026*	
H1B	0.3614	0.5884	0.2500	0.026*	
C2	0.41488 (18)	0.7186 (3)	0.28039 (9)	0.0188 (7)	
H2A	0.4693	0.6841	0.2796	0.023*	
H2B	0.4097	0.7771	0.2610	0.023*	
C3	0.3331 (2)	0.8365 (3)	0.31874 (10)	0.0253 (8)	
H3A	0.3358	0.8958	0.3003	0.038*	
H3B	0.3303	0.8679	0.3440	0.038*	
H3C	0.2843	0.7919	0.3123	0.038*	
C4	0.4799 (2)	0.8341 (3)	0.32982 (10)	0.0240 (7)	
H4A	0.4845	0.8938	0.3117	0.036*	
H4B	0.5289	0.7881	0.3305	0.036*	
H4C	0.4745	0.8649	0.3550	0.036*	
C5	0.16045 (19)	0.6242 (3)	0.37865 (9)	0.0203 (7)	
H5A	0.1270	0.6612	0.3580	0.024*	
H5B	0.1718	0.6772	0.3995	0.024*	
C6	0.11552 (19)	0.5262 (3)	0.39231 (10)	0.0234 (7)	
H6A	0.0666	0.5508	0.4045	0.028*	
H6B	0.0974	0.4787	0.3706	0.028*	
C7	0.1697 (2)	0.5093 (3)	0.45818 (10)	0.0283 (8)	
H7A	0.1147	0.5027	0.4668	0.042*	
H7B	0.1853	0.5868	0.4578	0.042*	
H7C	0.2085	0.4690	0.4754	0.042*	
C8	0.1435 (2)	0.3471 (3)	0.42013 (11)	0.0274 (8)	
H8A	0.0861	0.3437	0.4256	0.041*	
H8B	0.1769	0.3067	0.4396	0.041*	
H8C	0.1498	0.3141	0.3954	0.041*	
C9	0.24516 (18)	0.2700 (3)	0.31588 (9)	0.0180 (6)	
H9A	0.2240	0.2915	0.2901	0.022*	
H9B	0.2000	0.2358	0.3285	0.022*	
C10	0.31478 (18)	0.1882 (3)	0.31392 (9)	0.0175 (6)	
H10A	0.3235	0.1482	0.3381	0.021*	
H10B	0.3008	0.1341	0.2937	0.021*	
C11	0.3849 (2)	0.2949 (3)	0.26740 (9)	0.0238 (7)	
H11A	0.3777	0.2355	0.2489	0.036*	
H11B	0.4350	0.3355	0.2634	0.036*	
H11C	0.3380	0.3448	0.2644	0.036*	
C12	0.46108 (19)	0.1720 (3)	0.31080 (10)	0.0237 (7)	
H12A	0.4524	0.1108	0.2930	0.036*	
H12B	0.4661	0.1437	0.3367	0.036*	
H12C	0.5112	0.2110	0.3058	0.036*	
C13	0.37061 (18)	0.7122 (3)	0.42640 (9)	0.0166 (6)	
C14	0.3775 (2)	0.8151 (3)	0.45127 (10)	0.0294 (8)	
C15	0.36846 (19)	0.2779 (3)	0.42436 (9)	0.0194 (6)	
C16	0.3779 (2)	0.1874 (3)	0.45482 (10)	0.0252 (7)	
C17	0.55106 (18)	0.4851 (3)	0.34328 (8)	0.0157 (6)	
C18	0.6447 (2)	0.4702 (3)	0.34677 (10)	0.0244 (7)	
C19	0.5583 (2)	0.3809 (3)	0.44361 (11)	0.0290 (8)	
H19A	0.5641	0.3255	0.4236	0.035*	
H19B	0.5265	0.3485	0.4633	0.035*	
C20	0.6412 (2)	0.4186 (4)	0.46031 (11)	0.0331 (9)	
H20A	0.6634	0.3684	0.4805	0.040*	
H20B	0.6805	0.4245	0.4407	0.040*	
C21	0.6219 (2)	0.5295 (4)	0.47598 (13)	0.0426 (11)	
H21A	0.6701	0.5785	0.4767	0.051*	
H21B	0.6036	0.5231	0.5017	0.051*	
C22	0.5532 (2)	0.5721 (3)	0.44827 (11)	0.0330 (9)	
H22A	0.5109	0.6091	0.4620	0.040*	
H22B	0.5747	0.6252	0.4305	0.040*	
Cl1	0.13236 (5)	0.50928 (7)	0.27516 (2)	0.02051 (16)	
Cu1	0.23798 (2)	0.50916 (3)	0.317137 (10)	0.01044 (8)	
Cu2	0.39464 (2)	0.63387 (3)	0.350823 (10)	0.01304 (8)	
Cu3	0.28392 (2)	0.48008 (3)	0.401847 (10)	0.01366 (9)	
Cu4	0.39472 (2)	0.37149 (3)	0.343178 (10)	0.01343 (8)	
F1	0.38789 (19)	0.2286 (2)	0.48959 (7)	0.0616 (8)	
F2	0.44062 (16)	0.1223 (2)	0.45106 (7)	0.0499 (7)	
F3	0.31145 (16)	0.1262 (2)	0.45306 (9)	0.0618 (8)	
F4	0.45195 (17)	0.8352 (4)	0.46438 (13)	0.0738 (15)	0.894 (5)
F5	0.3505 (3)	0.9028 (2)	0.43088 (9)	0.0678 (12)	0.894 (5)
F6	0.3305 (2)	0.8121 (3)	0.47923 (9)	0.0526 (10)	0.894 (5)
F4B	0.418 (2)	0.8966 (17)	0.4377 (8)	0.0526 (10)	0.106 (5)
F5B	0.3162 (13)	0.855 (3)	0.4664 (11)	0.0738 (15)	0.106 (5)
F6B	0.420 (2)	0.788 (2)	0.4834 (6)	0.0678 (12)	0.106 (5)
F7	0.68329 (13)	0.5493 (2)	0.36747 (8)	0.0452 (7)	
F8	0.67280 (13)	0.4751 (2)	0.31263 (7)	0.0474 (7)	
F9	0.66948 (11)	0.37600 (18)	0.36251 (6)	0.0329 (5)	
N1	0.40701 (15)	0.7666 (2)	0.31829 (7)	0.0158 (5)	
N2	0.17040 (16)	0.4632 (2)	0.41990 (7)	0.0195 (6)	
N3	0.39102 (15)	0.2481 (2)	0.30606 (7)	0.0163 (5)	
O1	0.34445 (12)	0.56503 (18)	0.30425 (6)	0.0159 (4)	
O2	0.23492 (12)	0.58621 (18)	0.36547 (6)	0.0155 (4)	
O3	0.27429 (12)	0.36447 (17)	0.33646 (6)	0.0141 (4)	
O4	0.40721 (13)	0.72338 (18)	0.39660 (6)	0.0187 (5)	
O5	0.33254 (13)	0.6340 (2)	0.43810 (6)	0.0211 (5)	
O6	0.32578 (13)	0.35798 (19)	0.43373 (6)	0.0192 (5)	
O7	0.40130 (13)	0.25998 (19)	0.39509 (6)	0.0189 (5)	
O8	0.51284 (12)	0.39551 (19)	0.34027 (6)	0.0196 (5)	
O9	0.52741 (13)	0.58004 (19)	0.34305 (7)	0.0213 (5)	
O10	0.38614 (12)	0.49500 (16)	0.37767 (6)	0.0122 (4)	
H10	0.4325	0.4905	0.3976	0.018*	
O11	0.51928 (15)	0.4780 (2)	0.42819 (8)	0.0318 (6)	

### Atomic displacement parameters (Å^2^)

**Table:**

	*U*^11^	*U*^22^	*U*^33^	*U*^12^	*U*^13^	*U*^23^
C1	0.0238 (17)	0.0301 (18)	0.0103 (14)	−0.0031 (14)	0.0035 (12)	0.0015 (13)
C2	0.0169 (15)	0.0227 (17)	0.0180 (15)	0.0011 (12)	0.0087 (12)	0.0044 (13)
C3	0.0224 (17)	0.0220 (18)	0.0321 (19)	0.0076 (14)	0.0060 (14)	0.0069 (15)
C4	0.0229 (17)	0.0194 (17)	0.0296 (18)	−0.0061 (13)	0.0021 (14)	0.0040 (14)
C5	0.0201 (16)	0.0214 (17)	0.0199 (15)	0.0065 (13)	0.0046 (12)	0.0013 (13)
C6	0.0168 (15)	0.0282 (19)	0.0250 (17)	0.0009 (13)	0.0007 (13)	−0.0003 (14)
C7	0.0271 (17)	0.040 (2)	0.0189 (17)	−0.0023 (16)	0.0088 (14)	−0.0038 (15)
C8	0.0244 (18)	0.028 (2)	0.0312 (19)	−0.0064 (14)	0.0074 (15)	0.0034 (15)
C9	0.0167 (15)	0.0161 (15)	0.0212 (16)	−0.0021 (12)	0.0008 (12)	−0.0019 (13)
C10	0.0178 (15)	0.0175 (16)	0.0171 (15)	−0.0017 (12)	0.0009 (12)	−0.0018 (12)
C11	0.0274 (18)	0.0251 (18)	0.0197 (16)	−0.0027 (14)	0.0075 (14)	−0.0045 (14)
C12	0.0168 (16)	0.0243 (18)	0.0297 (18)	0.0062 (13)	0.0010 (13)	−0.0089 (15)
C13	0.0161 (15)	0.0173 (15)	0.0156 (15)	0.0034 (12)	−0.0033 (12)	−0.0047 (12)
C14	0.036 (2)	0.0260 (19)	0.0273 (19)	−0.0043 (16)	0.0099 (16)	−0.0086 (16)
C15	0.0195 (16)	0.0184 (16)	0.0197 (16)	−0.0030 (13)	−0.0031 (12)	0.0024 (13)
C16	0.035 (2)	0.0217 (18)	0.0199 (17)	0.0028 (15)	0.0053 (14)	0.0050 (14)
C17	0.0125 (13)	0.0211 (16)	0.0139 (14)	0.0018 (12)	0.0036 (11)	0.0025 (12)
C18	0.0166 (15)	0.0246 (18)	0.0324 (19)	0.0038 (13)	0.0054 (14)	0.0113 (15)
C19	0.0284 (19)	0.029 (2)	0.0283 (19)	0.0025 (15)	−0.0032 (15)	0.0047 (16)
C20	0.0233 (18)	0.048 (3)	0.0270 (19)	0.0037 (17)	−0.0060 (15)	0.0058 (18)
C21	0.032 (2)	0.049 (3)	0.044 (3)	−0.0038 (19)	−0.0155 (19)	−0.007 (2)
C22	0.029 (2)	0.030 (2)	0.037 (2)	0.0000 (16)	−0.0118 (16)	−0.0089 (17)
Cl1	0.0180 (3)	0.0238 (4)	0.0185 (4)	0.0001 (3)	−0.0054 (3)	0.0021 (3)
Cu1	0.01002 (16)	0.01276 (18)	0.00839 (16)	−0.00009 (13)	−0.00024 (12)	0.00033 (13)
Cu2	0.01405 (18)	0.01354 (18)	0.01150 (17)	−0.00088 (14)	0.00094 (13)	0.00082 (14)
Cu3	0.01306 (17)	0.01724 (19)	0.01080 (17)	0.00010 (14)	0.00167 (13)	0.00157 (14)
Cu4	0.01185 (18)	0.01471 (18)	0.01367 (17)	0.00029 (14)	0.00071 (13)	−0.00261 (14)
F1	0.115 (2)	0.0484 (17)	0.0214 (13)	0.0182 (16)	0.0036 (13)	0.0092 (12)
F2	0.0589 (17)	0.0486 (16)	0.0429 (15)	0.0237 (13)	0.0085 (12)	0.0191 (13)
F3	0.0555 (17)	0.0442 (17)	0.086 (2)	−0.0120 (13)	0.0071 (15)	0.0327 (16)
F4	0.0287 (16)	0.099 (3)	0.093 (3)	−0.0140 (17)	0.0005 (16)	−0.075 (3)
F5	0.121 (4)	0.0276 (16)	0.056 (2)	0.0143 (18)	0.012 (2)	−0.0113 (15)
F6	0.061 (2)	0.056 (2)	0.0442 (19)	−0.0053 (16)	0.0264 (16)	−0.0278 (16)
F4B	0.061 (2)	0.056 (2)	0.0442 (19)	−0.0053 (16)	0.0264 (16)	−0.0278 (16)
F5B	0.0287 (16)	0.099 (3)	0.093 (3)	−0.0140 (17)	0.0005 (16)	−0.075 (3)
F6B	0.121 (4)	0.0276 (16)	0.056 (2)	0.0143 (18)	0.012 (2)	−0.0113 (15)
F7	0.0210 (11)	0.0334 (13)	0.0787 (19)	−0.0063 (10)	−0.0113 (11)	0.0017 (13)
F8	0.0272 (12)	0.075 (2)	0.0427 (15)	0.0169 (12)	0.0203 (10)	0.0258 (13)
F9	0.0180 (10)	0.0303 (12)	0.0505 (14)	0.0051 (8)	0.0039 (9)	0.0172 (11)
N1	0.0137 (12)	0.0168 (13)	0.0173 (13)	0.0012 (10)	0.0034 (10)	0.0015 (10)
N2	0.0181 (13)	0.0260 (15)	0.0148 (13)	−0.0005 (11)	0.0039 (10)	0.0009 (11)
N3	0.0154 (12)	0.0155 (13)	0.0180 (13)	0.0011 (10)	0.0011 (10)	−0.0029 (11)
O1	0.0160 (11)	0.0204 (11)	0.0114 (10)	−0.0034 (9)	0.0026 (8)	0.0016 (9)
O2	0.0151 (10)	0.0186 (11)	0.0128 (10)	0.0018 (9)	0.0018 (8)	0.0002 (9)
O3	0.0128 (10)	0.0139 (10)	0.0153 (10)	0.0000 (8)	−0.0006 (8)	−0.0019 (8)
O4	0.0204 (11)	0.0191 (12)	0.0168 (11)	−0.0024 (9)	0.0022 (9)	−0.0016 (9)
O5	0.0227 (12)	0.0260 (13)	0.0147 (11)	−0.0046 (10)	0.0018 (9)	−0.0039 (10)
O6	0.0211 (12)	0.0200 (12)	0.0165 (11)	0.0013 (9)	0.0021 (9)	0.0037 (9)
O7	0.0222 (11)	0.0179 (11)	0.0167 (11)	0.0023 (9)	0.0020 (9)	0.0009 (9)
O8	0.0138 (11)	0.0213 (12)	0.0242 (12)	0.0000 (9)	0.0048 (9)	−0.0032 (10)
O9	0.0155 (11)	0.0201 (12)	0.0285 (13)	0.0027 (9)	0.0030 (9)	0.0045 (10)
O10	0.0122 (9)	0.0135 (10)	0.0109 (9)	−0.0005 (8)	0.0002 (7)	−0.0007 (8)
O11	0.0296 (14)	0.0249 (14)	0.0373 (15)	0.0007 (11)	−0.0193 (11)	−0.0005 (12)

### Geometric parameters (Å, °)

**Table:**

C1—O1	1.422 (4)	C14—F6	1.308 (4)
C1—C2	1.516 (5)	C14—F4B	1.310 (14)
C1—H1A	0.9900	C14—F6B	1.333 (15)
C1—H1B	0.9900	C14—F5	1.346 (5)
C2—N1	1.484 (4)	C15—O7	1.230 (4)
C2—H2A	0.9900	C15—O6	1.263 (4)
C2—H2B	0.9900	C15—C16	1.545 (5)
C3—N1	1.485 (4)	C16—F2	1.316 (4)
C3—H3A	0.9800	C16—F3	1.319 (4)
C3—H3B	0.9800	C16—F1	1.332 (4)
C3—H3C	0.9800	C17—O9	1.221 (4)
C4—N1	1.484 (4)	C17—O8	1.260 (4)
C4—H4A	0.9800	C17—C18	1.545 (4)
C4—H4B	0.9800	C18—F9	1.326 (4)
C4—H4C	0.9800	C18—F8	1.334 (4)
C5—O2	1.423 (3)	C18—F7	1.340 (4)
C5—C6	1.505 (5)	C19—O11	1.434 (4)
C5—H5A	0.9900	C19—C20	1.512 (5)
C5—H5B	0.9900	C19—H19A	0.9900
C6—N2	1.490 (4)	C19—H19B	0.9900
C6—H6A	0.9900	C20—C21	1.506 (6)
C6—H6B	0.9900	C20—H20A	0.9900
C7—N2	1.473 (4)	C20—H20B	0.9900
C7—H7A	0.9800	C21—C22	1.528 (5)
C7—H7B	0.9800	C21—H21A	0.9900
C7—H7C	0.9800	C21—H21B	0.9900
C8—N2	1.483 (4)	C22—O11	1.439 (4)
C8—H8A	0.9800	C22—H22A	0.9900
C8—H8B	0.9800	C22—H22B	0.9900
C8—H8C	0.9800	Cl1—Cu1	2.1948 (8)
C9—O3	1.426 (4)	Cu1—O2	1.962 (2)
C9—C10	1.523 (4)	Cu1—O1	1.966 (2)
C9—H9A	0.9900	Cu1—O3	1.968 (2)
C9—H9B	0.9900	Cu2—O10	1.954 (2)
C10—N3	1.496 (4)	Cu2—O4	1.956 (2)
C10—H10A	0.9900	Cu2—O1	1.975 (2)
C10—H10B	0.9900	Cu2—N1	2.009 (3)
C11—N3	1.484 (4)	Cu2—O9	2.317 (2)
C11—H11A	0.9800	Cu3—O2	1.956 (2)
C11—H11B	0.9800	Cu3—O10	1.957 (2)
C11—H11C	0.9800	Cu3—O6	1.961 (2)
C12—N3	1.478 (4)	Cu3—N2	2.032 (3)
C12—H12A	0.9800	Cu3—O5	2.377 (2)
C12—H12B	0.9800	Cu4—O10	1.954 (2)
C12—H12C	0.9800	Cu4—O8	1.974 (2)
C13—O5	1.231 (4)	Cu4—O3	1.976 (2)
C13—O4	1.267 (4)	Cu4—N3	1.998 (3)
C13—C14	1.533 (5)	Cu4—O7	2.287 (2)
C14—F5B	1.278 (15)	O10—H10	0.9990
C14—F4	1.296 (4)		
			
O1—C1—C2	109.0 (2)	F8—C18—C17	109.7 (3)
O1—C1—H1A	109.9	F7—C18—C17	112.5 (3)
C2—C1—H1A	109.9	O11—C19—C20	105.1 (3)
O1—C1—H1B	109.9	O11—C19—H19A	110.7
C2—C1—H1B	109.9	C20—C19—H19A	110.7
H1A—C1—H1B	108.3	O11—C19—H19B	110.7
N1—C2—C1	109.5 (2)	C20—C19—H19B	110.7
N1—C2—H2A	109.8	H19A—C19—H19B	108.8
C1—C2—H2A	109.8	C21—C20—C19	102.0 (3)
N1—C2—H2B	109.8	C21—C20—H20A	111.4
C1—C2—H2B	109.8	C19—C20—H20A	111.4
H2A—C2—H2B	108.2	C21—C20—H20B	111.4
N1—C3—H3A	109.5	C19—C20—H20B	111.4
N1—C3—H3B	109.5	H20A—C20—H20B	109.2
H3A—C3—H3B	109.5	C20—C21—C22	103.5 (3)
N1—C3—H3C	109.5	C20—C21—H21A	111.1
H3A—C3—H3C	109.5	C22—C21—H21A	111.1
H3B—C3—H3C	109.5	C20—C21—H21B	111.1
N1—C4—H4A	109.5	C22—C21—H21B	111.1
N1—C4—H4B	109.5	H21A—C21—H21B	109.0
H4A—C4—H4B	109.5	O11—C22—C21	106.5 (3)
N1—C4—H4C	109.5	O11—C22—H22A	110.4
H4A—C4—H4C	109.5	C21—C22—H22A	110.4
H4B—C4—H4C	109.5	O11—C22—H22B	110.4
O2—C5—C6	107.8 (3)	C21—C22—H22B	110.4
O2—C5—H5A	110.1	H22A—C22—H22B	108.6
C6—C5—H5A	110.1	O2—Cu1—O1	97.18 (9)
O2—C5—H5B	110.1	O2—Cu1—O3	98.75 (9)
C6—C5—H5B	110.1	O1—Cu1—O3	98.13 (9)
H5A—C5—H5B	108.5	O2—Cu1—Cl1	121.31 (6)
N2—C6—C5	109.6 (3)	O1—Cu1—Cl1	120.73 (7)
N2—C6—H6A	109.8	O3—Cu1—Cl1	116.00 (6)
C5—C6—H6A	109.8	O10—Cu2—O4	94.81 (9)
N2—C6—H6B	109.8	O10—Cu2—O1	89.98 (9)
C5—C6—H6B	109.8	O4—Cu2—O1	160.49 (9)
H6A—C6—H6B	108.2	O10—Cu2—N1	173.56 (9)
N2—C7—H7A	109.5	O4—Cu2—N1	91.20 (10)
N2—C7—H7B	109.5	O1—Cu2—N1	85.10 (10)
H7A—C7—H7B	109.5	O10—Cu2—O9	85.32 (8)
N2—C7—H7C	109.5	O4—Cu2—O9	102.75 (9)
H7A—C7—H7C	109.5	O1—Cu2—O9	96.48 (8)
H7B—C7—H7C	109.5	N1—Cu2—O9	91.08 (9)
N2—C8—H8A	109.5	O2—Cu3—O10	88.29 (8)
N2—C8—H8B	109.5	O2—Cu3—O6	172.03 (9)
H8A—C8—H8B	109.5	O10—Cu3—O6	92.93 (9)
N2—C8—H8C	109.5	O2—Cu3—N2	86.38 (10)
H8A—C8—H8C	109.5	O10—Cu3—N2	172.39 (10)
H8B—C8—H8C	109.5	O6—Cu3—N2	91.62 (10)
O3—C9—C10	109.3 (2)	O2—Cu3—O5	86.47 (9)
O3—C9—H9A	109.8	O10—Cu3—O5	84.38 (8)
C10—C9—H9A	109.8	O6—Cu3—O5	101.48 (9)
O3—C9—H9B	109.8	N2—Cu3—O5	100.69 (10)
C10—C9—H9B	109.8	O10—Cu4—O8	92.30 (9)
H9A—C9—H9B	108.3	O10—Cu4—O3	89.22 (8)
N3—C10—C9	109.5 (3)	O8—Cu4—O3	168.40 (9)
N3—C10—H10A	109.8	O10—Cu4—N3	173.88 (9)
C9—C10—H10A	109.8	O8—Cu4—N3	93.10 (10)
N3—C10—H10B	109.8	O3—Cu4—N3	84.91 (9)
C9—C10—H10B	109.8	O10—Cu4—O7	87.29 (8)
H10A—C10—H10B	108.2	O8—Cu4—O7	98.53 (9)
N3—C11—H11A	109.5	O3—Cu4—O7	93.03 (8)
N3—C11—H11B	109.5	N3—Cu4—O7	94.73 (9)
H11A—C11—H11B	109.5	C4—N1—C2	110.0 (2)
N3—C11—H11C	109.5	C4—N1—C3	108.8 (3)
H11A—C11—H11C	109.5	C2—N1—C3	111.6 (2)
H11B—C11—H11C	109.5	C4—N1—Cu2	113.88 (19)
N3—C12—H12A	109.5	C2—N1—Cu2	103.03 (19)
N3—C12—H12B	109.5	C3—N1—Cu2	109.54 (19)
H12A—C12—H12B	109.5	C7—N2—C8	109.5 (3)
N3—C12—H12C	109.5	C7—N2—C6	111.2 (3)
H12A—C12—H12C	109.5	C8—N2—C6	109.3 (3)
H12B—C12—H12C	109.5	C7—N2—Cu3	109.3 (2)
O5—C13—O4	130.9 (3)	C8—N2—Cu3	112.3 (2)
O5—C13—C14	117.0 (3)	C6—N2—Cu3	105.17 (19)
O4—C13—C14	112.1 (3)	C12—N3—C11	109.9 (2)
F4—C14—F6	109.3 (4)	C12—N3—C10	109.2 (3)
F5B—C14—F4B	108 (2)	C11—N3—C10	111.5 (2)
F5B—C14—F6B	96 (2)	C12—N3—Cu4	114.56 (19)
F4B—C14—F6B	105 (2)	C11—N3—Cu4	108.6 (2)
F4—C14—F5	107.6 (4)	C10—N3—Cu4	103.00 (18)
F6—C14—F5	104.0 (3)	C1—O1—Cu1	119.59 (18)
F4—C14—C13	112.7 (3)	C1—O1—Cu2	112.44 (19)
F6—C14—C13	113.2 (3)	Cu1—O1—Cu2	105.70 (9)
F4B—C14—C13	115.1 (10)	C5—O2—Cu3	108.43 (17)
F6B—C14—C13	107.4 (12)	C5—O2—Cu1	121.87 (18)
F5—C14—C13	109.4 (3)	Cu3—O2—Cu1	102.93 (10)
O7—C15—O6	130.7 (3)	C9—O3—Cu1	117.73 (17)
O7—C15—C16	116.1 (3)	C9—O3—Cu4	112.83 (17)
O6—C15—C16	113.2 (3)	Cu1—O3—Cu4	106.00 (10)
F2—C16—F3	107.9 (3)	C13—O4—Cu2	127.6 (2)
F2—C16—F1	106.5 (3)	C13—O5—Cu3	125.9 (2)
F3—C16—F1	107.2 (3)	C15—O6—Cu3	127.6 (2)
F2—C16—C15	113.2 (3)	C15—O7—Cu4	125.6 (2)
F3—C16—C15	109.6 (3)	C17—O8—Cu4	127.6 (2)
F1—C16—C15	112.2 (3)	C17—O9—Cu2	124.8 (2)
O9—C17—O8	131.7 (3)	Cu2—O10—Cu4	110.45 (10)
O9—C17—C18	115.2 (3)	Cu2—O10—Cu3	113.15 (10)
O8—C17—C18	113.1 (3)	Cu4—O10—Cu3	108.20 (9)
F9—C18—F8	107.8 (3)	Cu2—O10—H10	108.2
F9—C18—F7	106.0 (3)	Cu4—O10—H10	108.3
F8—C18—F7	106.5 (3)	Cu3—O10—H10	108.4
F9—C18—C17	113.9 (3)	C19—O11—C22	109.0 (3)
			
O1—C1—C2—N1	−45.7 (3)	O10—Cu3—O2—C5	−165.02 (19)
O2—C5—C6—N2	52.7 (3)	N2—Cu3—O2—C5	20.4 (2)
O3—C9—C10—N3	−42.2 (3)	O5—Cu3—O2—C5	−80.55 (19)
O5—C13—C14—F5B	−46 (3)	O10—Cu3—O2—Cu1	64.62 (9)
O4—C13—C14—F5B	135 (3)	N2—Cu3—O2—Cu1	−109.95 (11)
O5—C13—C14—F4	114.5 (4)	O5—Cu3—O2—Cu1	149.09 (9)
O4—C13—C14—F4	−64.8 (5)	O1—Cu1—O2—C5	146.6 (2)
O5—C13—C14—F6	−10.3 (5)	O3—Cu1—O2—C5	−113.9 (2)
O4—C13—C14—F6	170.4 (3)	Cl1—Cu1—O2—C5	13.8 (2)
O5—C13—C14—F4B	−179.5 (19)	O1—Cu1—O2—Cu3	−91.71 (10)
O4—C13—C14—F4B	1.2 (19)	O3—Cu1—O2—Cu3	7.71 (10)
O5—C13—C14—F6B	63.9 (19)	Cl1—Cu1—O2—Cu3	135.49 (6)
O4—C13—C14—F6B	−115.4 (19)	C10—C9—O3—Cu1	138.4 (2)
O5—C13—C14—F5	−125.8 (4)	C10—C9—O3—Cu4	14.3 (3)
O4—C13—C14—F5	54.9 (4)	O2—Cu1—O3—C9	146.95 (19)
O7—C15—C16—F2	21.6 (4)	O1—Cu1—O3—C9	−114.43 (19)
O6—C15—C16—F2	−160.1 (3)	Cl1—Cu1—O3—C9	15.7 (2)
O7—C15—C16—F3	−98.9 (4)	O2—Cu1—O3—Cu4	−85.68 (10)
O6—C15—C16—F3	79.5 (4)	O1—Cu1—O3—Cu4	12.93 (11)
O7—C15—C16—F1	142.1 (3)	Cl1—Cu1—O3—Cu4	143.02 (6)
O6—C15—C16—F1	−39.5 (4)	O10—Cu4—O3—C9	−171.33 (19)
O9—C17—C18—F9	153.1 (3)	O8—Cu4—O3—C9	91.0 (5)
O8—C17—C18—F9	−28.5 (4)	N3—Cu4—O3—C9	10.4 (2)
O9—C17—C18—F8	−86.0 (4)	O7—Cu4—O3—C9	−84.08 (19)
O8—C17—C18—F8	92.4 (3)	O10—Cu4—O3—Cu1	58.43 (10)
O9—C17—C18—F7	32.4 (4)	O8—Cu4—O3—Cu1	−39.2 (5)
O8—C17—C18—F7	−149.2 (3)	N3—Cu4—O3—Cu1	−119.84 (11)
O11—C19—C20—C21	36.9 (4)	O7—Cu4—O3—Cu1	145.67 (9)
C19—C20—C21—C22	−33.5 (4)	O5—C13—O4—Cu2	17.2 (5)
C20—C21—C22—O11	19.0 (4)	C14—C13—O4—Cu2	−163.6 (2)
C1—C2—N1—C4	169.3 (3)	O10—Cu2—O4—C13	−39.4 (3)
C1—C2—N1—C3	−69.9 (3)	O1—Cu2—O4—C13	64.3 (4)
C1—C2—N1—Cu2	47.6 (3)	N1—Cu2—O4—C13	142.9 (3)
O4—Cu2—N1—C4	51.0 (2)	O9—Cu2—O4—C13	−125.7 (3)
O1—Cu2—N1—C4	−148.2 (2)	O4—C13—O5—Cu3	−15.7 (5)
O9—Cu2—N1—C4	−51.8 (2)	C14—C13—O5—Cu3	165.1 (2)
O4—Cu2—N1—C2	170.02 (17)	O2—Cu3—O5—C13	−51.2 (3)
O1—Cu2—N1—C2	−29.17 (17)	O10—Cu3—O5—C13	37.4 (3)
O9—Cu2—N1—C2	67.24 (17)	O6—Cu3—O5—C13	129.2 (3)
O4—Cu2—N1—C3	−71.1 (2)	N2—Cu3—O5—C13	−136.9 (3)
O1—Cu2—N1—C3	89.7 (2)	O7—C15—O6—Cu3	8.0 (5)
O9—Cu2—N1—C3	−173.9 (2)	C16—C15—O6—Cu3	−170.1 (2)
C5—C6—N2—C7	84.5 (3)	O10—Cu3—O6—C15	−41.7 (3)
C5—C6—N2—C8	−154.5 (3)	N2—Cu3—O6—C15	132.2 (3)
C5—C6—N2—Cu3	−33.7 (3)	O5—Cu3—O6—C15	−126.6 (3)
O2—Cu3—N2—C7	−111.5 (2)	O6—C15—O7—Cu4	−1.2 (5)
O6—Cu3—N2—C7	76.2 (2)	C16—C15—O7—Cu4	176.9 (2)
O5—Cu3—N2—C7	−25.8 (2)	O10—Cu4—O7—C15	30.6 (3)
O2—Cu3—N2—C8	126.7 (2)	O8—Cu4—O7—C15	122.6 (3)
O6—Cu3—N2—C8	−45.5 (2)	O3—Cu4—O7—C15	−58.4 (3)
O5—Cu3—N2—C8	−147.5 (2)	N3—Cu4—O7—C15	−143.6 (3)
O2—Cu3—N2—C6	7.9 (2)	O9—C17—O8—Cu4	−12.4 (5)
O6—Cu3—N2—C6	−164.4 (2)	C18—C17—O8—Cu4	169.5 (2)
O5—Cu3—N2—C6	93.6 (2)	O10—Cu4—O8—C17	−30.2 (3)
C9—C10—N3—C12	170.1 (3)	O3—Cu4—O8—C17	67.2 (6)
C9—C10—N3—C11	−68.2 (3)	N3—Cu4—O8—C17	146.9 (3)
C9—C10—N3—Cu4	48.0 (3)	O7—Cu4—O8—C17	−117.8 (3)
O8—Cu4—N3—C12	41.2 (2)	O8—C17—O9—Cu2	15.4 (5)
O3—Cu4—N3—C12	−150.3 (2)	C18—C17—O9—Cu2	−166.5 (2)
O7—Cu4—N3—C12	−57.7 (2)	O10—Cu2—O9—C17	24.9 (3)
O8—Cu4—N3—C11	−82.1 (2)	O4—Cu2—O9—C17	118.8 (3)
O3—Cu4—N3—C11	86.44 (19)	O1—Cu2—O9—C17	−64.6 (3)
O7—Cu4—N3—C11	179.06 (19)	N1—Cu2—O9—C17	−149.8 (3)
O8—Cu4—N3—C10	159.62 (18)	O4—Cu2—O10—Cu4	−168.32 (10)
O3—Cu4—N3—C10	−31.84 (18)	O1—Cu2—O10—Cu4	30.61 (10)
O7—Cu4—N3—C10	60.78 (18)	O9—Cu2—O10—Cu4	−65.89 (10)
C2—C1—O1—Cu1	144.4 (2)	O4—Cu2—O10—Cu3	70.20 (11)
C2—C1—O1—Cu2	19.6 (3)	O1—Cu2—O10—Cu3	−90.86 (11)
O2—Cu1—O1—C1	−115.4 (2)	O9—Cu2—O10—Cu3	172.64 (11)
O3—Cu1—O1—C1	144.7 (2)	O8—Cu4—O10—Cu2	72.73 (11)
Cl1—Cu1—O1—C1	17.8 (2)	O3—Cu4—O10—Cu2	−95.76 (10)
O2—Cu1—O1—Cu2	12.61 (11)	O7—Cu4—O10—Cu2	171.17 (10)
O3—Cu1—O1—Cu2	−87.35 (10)	O8—Cu4—O10—Cu3	−162.91 (10)
Cl1—Cu1—O1—Cu2	145.78 (6)	O3—Cu4—O10—Cu3	28.60 (10)
O10—Cu2—O1—C1	−170.14 (19)	O7—Cu4—O10—Cu3	−64.47 (10)
O4—Cu2—O1—C1	85.4 (3)	O2—Cu3—O10—Cu2	23.58 (11)
N1—Cu2—O1—C1	5.7 (2)	O6—Cu3—O10—Cu2	−164.31 (11)
O9—Cu2—O1—C1	−84.9 (2)	O5—Cu3—O10—Cu2	−63.05 (10)
O10—Cu2—O1—Cu1	57.71 (10)	O2—Cu3—O10—Cu4	−99.15 (10)
O4—Cu2—O1—Cu1	−46.8 (3)	O6—Cu3—O10—Cu4	72.97 (11)
N1—Cu2—O1—Cu1	−126.46 (11)	O5—Cu3—O10—Cu4	174.22 (10)
O9—Cu2—O1—Cu1	143.00 (10)	C20—C19—O11—C22	−25.9 (4)
C6—C5—O2—Cu3	−44.1 (3)	C21—C22—O11—C19	4.2 (4)
C6—C5—O2—Cu1	74.9 (3)		

### Hydrogen-bond geometry (Å, °)

**Table:**

*D*—H···*A*	*D*—H	H···*A*	*D*···*A*	*D*—H···*A*
O10—H10···O11	1.00	1.73	2.723 (3)	174
